# Limitations of Explainability for Established Prognostic Biomarkers of Prostate Cancer

**DOI:** 10.3389/fgene.2021.649429

**Published:** 2021-07-22

**Authors:** Kalifa Manjang, Olli Yli-Harja, Matthias Dehmer, Frank Emmert-Streib

**Affiliations:** ^1^Predictive Society and Data Analytics Lab, Faculty of Information Technology and Communication Sciences, Tampere University, Tampere, Finland; ^2^Computational Systems Biology, Tampere University, Tampere, Finland; ^3^Institute for Systems Biology, Seattle, WA, United States; ^4^Faculty of Medicine and Health Technology, Institute of Biosciences and Medical Technology, Tampere University, Tampere, Finland; ^5^Department of Computer Science, Swiss Distance University of Applied Sciences, Brig, Switzerland; ^6^Department of Mechatronics and Biomedical Computer Science, University for Health Sciences, Medical Informatics and Technology (UMIT), Hall, Austria; ^7^College of Artificial Intelligence, Nankai University, Tianjin, China

**Keywords:** prostate cancer, biomarkers, prognostic biomarkers, survival analysis, data science, computational biology, biostatistics

## Abstract

High-throughput technologies do not only provide novel means for basic biological research but also for clinical applications in hospitals. For instance, the usage of gene expression profiles as prognostic biomarkers for predicting cancer progression has found widespread interest. Aside from predicting the progression of patients, it is generally believed that such prognostic biomarkers also provide valuable information about disease mechanisms and the underlying molecular processes that are causal for a disorder. However, the latter assumption has been challenged. In this paper, we study this problem for prostate cancer. Specifically, we investigate a large number of previously published prognostic signatures of prostate cancer based on gene expression profiles and show that none of these can provide unique information about the underlying disease etiology of prostate cancer. Hence, our analysis reveals that none of the studied signatures has a sensible biological meaning. Overall, this shows that all studied prognostic signatures are merely black-box models allowing sensible predictions of prostate cancer outcome but are not capable of providing causal explanations to enhance the understanding of prostate cancer.

## 1. Introduction

Prostate cancer (PCa) is the second most prevalent cancer among men, the average age of diagnosis is 66 years, and about 60% of diagnosed cases occur in men over 65 years old. In the United States, for example, 191, 930 newly diagnosis cases of PCa are estimated in 2020, resulting in about 33, 330 mortalities (Siegel et al., 2020). A substantial proportion of PCa is characterized as slow-growing and indolent requiring no immediate therapeutic intervention. However, tumor stages T1 and T2, and tumor stages higher than T2 are more aggressive and invade the surrounding organs and the patient is more likely to die from the disease (Chen et al., 2020). Specifically, for men with local or regional PCa, the 5-year survival rate is almost 100%, whereas the 5-year survival rate for men with metastatic PCa is 31%.

Since the inception of high-throughput technologies, a large number of molecular markers have been described in the literature capable of distinguishing cancer patients with good and bad prognosis. Nonetheless, few found their way into clinical decision making. Many biomarker studies have used genome-wide gene expression analysis to define unique gene expression signatures related to the prognosis of PCa. For example, Chen et al. (2012) developed a 7-gene prognostic signature through a cluster-correlation analysis to identify differentially expressed genes in various cell types associated with PCa progression. Likewise, in Liu et al. (2007), the gene expression of CD44+CD24 of low tumorigenic breast and normal breast epithelium cells were compared. They used the differentially expressed genes to construct a 186-gene “invasiveness” gene signature. The signatures were tested for their association with two clinical endpoints, overall survival and metastasis-free survival, in breast and other cancer patients. Interestingly, the signature was substantially correlated with the two survival endpoints in patients with breast cancer and other types of cancer. Another study by Ramaswamy et al. (2003) examined the molecular variations between human primary tumors and metastases. The gene expression profiles of different types of adenocarcinoma metastases and unmatched primary adenocarcinomas were compared, and the analysis identified a gene expression signature capable of separating primary from metastatic adenocarcinomas (Ramaswamy et al., 2003).

There are also studies that use more advanced approaches to derive the gene signatures. In a study by Irshad et al. (2013), a 19-gene signature enriched in indolent prostate tumors was identified. Their final signature includes three genes that, through a further classification of the 19-gene signature, was established by a decision tree (DT) model. Similarly, a combination of artificial neural network analysis and data from literature search and other studies resulted in a panel of PCa progression markers, which were used in a transcriptomic analysis of 29 radical prostatectomy samples correlated with clinical outcome (Larkin et al., 2012).

Aside from such potential success stories, there are several well-known problems with prognostic signatures. One such problem relates to the stability of the selection of prognostic genes. In Michiels et al. (2005), this has been studied for various cancer types and the authors found that the size of the training data as well as the patient data both crucially effect the selection of such genes. For breast cancer, this effect has been quantified by Ein-Dor et al. (2006). Specifically, the authors showed that thousands of patient samples are needed for achieving an overlap of 50% between two predictive sets of prognostic genes. Further examples of such studies reporting similar results can be found in Kim (2009), Haury et al. (2011), and Gilhodes et al. (2017). A well-recognized study by Venet et al. (2011) addressed yet another problem by showing that many random breast cancer gene sets have similar prognostic prediction capabilities as biomarker (BM) signatures. The study by Goh and Wong (2018) extended this by removing proliferation genes. A conceptual problem of both studies is that random gene sets could still share biological similarity on the level of biological processes (BPs). The reason for this is that no systematic mechanism has been implemented that would eliminate such a similarity. In contrast, the study by Manjang et al. (2021) introduced a gene removal procedure (GRP) that accomplished this.

The purpose of this paper is to test a hypothesis about the systems behavior of PCa. Specifically, despite well-documented differences between breast cancer and PCa, e.g., PCa affects men exclusively, whereas breast cancer commonly affects women, likewise both tumors arise in different organs involving different physiological functions, we hypothesize that their functional similarity, e.g., via the hallmarks of cancer (Hanahan and Weinberg, 2000, 2011), induces similar results for prognostic signatures. In order to investigate this, we study 32 published prognostic PCa signatures from the literature and demonstrate that random gene sets can be found with similar prediction capabilities as these signatures but opposite biological meaning.

The paper is organized as follows. In the next section, we describe our methods and data used for our analysis. Then we present and discuss our results. The paper completes with concluding remarks.

## 2. Materials and Methods

In this section, we provide information about the data and methods used for our analysis.

### 2.1. Biomarker Signatures

We identified reported PCa gene signatures from a literature search. From this search, we found 32 signatures from 31 studies that have been published between 2002 and 2020. For all signatures, the Entrez gene IDs corresponding to the HGNC gene symbols are determined. All genes without an associated Entrez gene ID are discarded. Table 1 shows an overview of the published gene signatures we use for our study.

**Table 1:** Overview of published and evaluated prognostic signatures for prostate cancer used for our study.

**Acronym of a study**	Number of genes^*^	**Cancer type**	**Reference**
AGELL	12	Prostate cancer	Agell et al., 2012
BIBIKOVA	16	Prostate cancer	Bibikova et al., 2007
BISMAR	12	Prostate cancer	Bismar et al., 2006
CHEN	4	Prostate cancer	Chen et al., 2020
CHEN_CC	7	Prostate cancer	Chen et al., 2012
CHEVILLE	2	Prostate cancer	Cheville et al., 2008
CHU	8	Prostate cancer	Chu et al., 2018
CUZICK	31	Prostate cancer	Cuzick et al., 2011
GLINSKY	11	Multiple cancers	Glinsky et al., 2005
IRSHAD	19	Prostate cancer	Irshad et al., 2013
IRSHAD_1	3	Prostate cancer	Irshad et al., 2013
LARKIN	7	Prostate cancer	Larkin et al., 2012
LI	6	Prostate cancer	Li et al., 2019
LIU	167	Multiple cancers	Liu et al., 2007
LONG	12	Prostate cancer	Long et al., 2011
NAKAGAWA	17	Prostate cancer	Nakagawa et al., 2008
PENNEY	157	Prostate cancer	Penney et al., 2011
RAMASWAMY	16	Multiple cancers	Ramaswamy et al., 2003
REDDY	16	Prostate cancer	Reddy and Balk, 2006
ROSS-ADAMS	100	Prostate cancer	Ross-Adams et al., 2015
ROSS	6	Prostate cancer	Ross et al., 2012
SAAL	162	Multiple cancers	Saal et al., 2007
SHARMA	15	Prostate cancer	Sharma et al., 2013
SINGH	5	Prostate cancer	Singh et al., 2002
SONG	15	Prostate cancer	Song et al., 2019
STEPHENSON	10	Prostate cancer	Stephenson et al., 2005
TALANTOV	3	Prostate cancer	Talantov et al., 2010
TANDEFELT	36	Prostate cancer	Tandefelt et al., 2013
TRUE	86	Prostate cancer	True et al., 2006
WANG	43	Prostate cancer	Wang et al., 2017
WU	29	Prostate cancer	Wu et al., 2013
YU	14	Multiple cancers	Yu et al., 2007

Number of genes^*^ corresponds to the number of genes in a signature after conversion from HGNC gene symbols to Entrez gene IDs.

### 2.2. Gene Expression Data

We collected RNA-seq data (HTSeq-FPKM and HTSeq-FPKM-UQ) of patients with PCa from the TCGA-PRAD project. We obtained the data from the UCSC Xena GDC data hub (https://xenabrowser.net/datapages/) on September 7, 2020. FPKM stands for *Fragments Per Kilobase of transcript per Million* mapped reads (Trapnell et al., 2010). It accounts for a situation in which only 1 end of a pair-end read is mapped. The FPKM of a gene is estimated as follows:

(1) $$\begin{array}{l} FPKM\\ =\frac{10^{9}\text{× number of reads mapped to the gene}}{\begin{array}{l} (\text{number of reads mapped to all protein}-\text{coding genes}\\ \;\times \text{length of the gene in base pairs}) \end{array}} \end{array}$$

Similarly, FPKM-UQ means Fragments Per Kilobase of transcript per Million mapped reads upper quartile. It is a modified estimate of FPKM where the total protein-coding read count is replaced by the 75th percentile read count for a sample. A notable difference between the two is the values of FPKM-UQ tends to be much higher due to the significant disparity between the total mapped number of reads in an alignment and the mapped number of reads to one gene.

The gene expression data set used in our study contains 551 samples, of which 498 are primary solid tumors, 52 are solid tissue normal, and one is metastatic. We exclude the metastatic and solid tissue normal samples from the data set. From these data, we used only protein-coding genes without missing information for the HTSeq-FPKM data cohort. Likewise, from the HTSeq-FPKM-UQ data we used only genes with <2% missing information across all samples. The final HTSeq-FPKM data set contains 498 samples and 16, 428 genes, whereas the HTSeq-FPKM-UQ data set contains 498 samples and 15, 165 genes. Lastly, patient survival information for each sample was derived from Liu et al. (2018). Specifically, we used the progression-free interval end-points. In this paper, we refer to the HTseq-FPKM and HTSeq-FPKM-UQ in our analysis as GDC cohort A and GDC cohort B, respectively.

### 2.3. Outcome Association

In order to determine the prognostic importance of a random gene set, we perform a survival analysis. We estimate Kaplan–Meier survival curves and compare these with a Mantel–Haenszel test (Emmert-Streib and Dehmer, 2019). That means each comparison provides a *p*-value from such a hypothesis test.

The patients are stratified into two classes (low and high risk) by using the PC1 method. This method categorizes patients according to a particular gene set. Hence, the resulting survival analysis is a function of the gene set used to categorize the patients. Overall, our study consists of three main steps: first, the selection/construction of random gene set; second, the classification of patients samples; and third, the survival analysis.

In the next section, we explain our method we use as GRP for constructing random gene sets.

### 2.4. Gene Removal Procedure

Our GRP entails the removal of both the BM signatures and genes that belong to the same BPs as the genes in the BM signatures. The gene ontology (GO) is hierarchical (Ashburner et al., 2000). Hence, we approach this analysis iteratively by removing genes of BPs successively from the same hierarchy level. The GRP we use is defined as follows:

1. G: the genes in the PCa data set (16, 425 and 15, 165 for GDC cohort A and B, respectively).
2. *BM*_*i*_:*g*_*i*_, …, *g*_*m*_. *BM*_*i*_ is the gene signature *i* (*i* range from 1 to 32) and *g*_*i*_, …, *g*_*m*_ are the genes in the respective signatures.
3. Removing biomarker genes in signature *BM*_*i*_ from G. This produces a new set of genes $G_{i}^{\prime }$ with $G_{i}^{\prime }=G\backslash BM_{i}$.
4. 3^*^ Optional step: Remove the proliferation genes, PG from G. This gives a new set of genes $G_{i}^{\prime *}$ with $G_{i}^{\prime *}=G^{\prime }\backslash PG$.
5. Map the genes in *BM*_*i*_ to GO-terms and the corresponding hierarchy levels. This gives: *BM*_*i*_ = {*g*_*i*_, …, *g*_*m*_} → *R* = {(*GO*_1_, *L*_1_), …, (*GO*_*t*_, *L*_*t*_)}(Manjang et al., 2020).
  Note, each gene can be connected to more than one GO-term. For this reason, *m* ≤ *t*.
6. Map each GO-term in R, i.e., *GO*_*i*_ with *i*∈{1, …, *t*}, to its gene set *GS*_*i*_.
7. For each biomarker set *i*: Loop-over the elements in set *R*.
  a. Delete all the genes associated with the GO-terms in set *R*. This results in a new set given by *G*^′′^ = *G*′\*D*, where *D* is the set of genes having GO-terms in *R*, i.e., $D=\cup_{i=1}^{t}GS_{i}$.
8. From *G*^′′^, we loop from 1 to 1, 000:
  a. We sample new sets of biomarker genes of size |*BM*_*i*_∈*G*| and perform the prognostic task. We repeat this for 1, 000 times.
  b. Application of a Bonferroni correction to the *p*-values.
  c. Set *G*′ = *G*^′′^. Stop if *l* = *L*_*min*_(*i*) or $\left|G^{\prime \prime }|<2\times |BM_{i}\in G\right|$.

In the above procedure, the optional step called 3^*^ involves the removal of the 664 genes that are related to proliferation (this gene set is called PG). We extracted the genes in PG from Goh and Wong (2018).

The prediction results are assessed using the *p*-values obtained from the survival analysis. We call a random gene set with a significant *p*-value, a *surrogate gene set*.

### 2.5. Unsupervised Classification

The patient samples are categorized using the PC1 stratification method, which is based on a principal component analysis (PCA). Briefly, PCA is a dimension reduction technique (this involves reducing the size of the data set). The goal is to transform a large data set into a smaller ones having a lower dimensional representation. This method trades a little accuracy for simplicity, thus achieving interpretability as well as minimal loss of information (Lever et al., 2017). For performing the PC1 method, we use the R function "prcomp" to obtain the first principal component (PC1) of a signature. The patients are then divided into two groups according to the median of the PC1, i.e., a sample is either categorized as group −1 if the PC1 is below the median or as group +1 if the PC1 is above the median value. Hence, the PC1 method is used to classify (or group) the patients into two classes, whereas this separation depends on a signature gene set. This approach has been previously used (see, e.g., Venet et al., 2011).

Formally, our analysis is based on a gene expression matrix of the form *X*∈ℝ^*m*^ × ℝ^*n*^, where *m* is the number of genes and *n* is the number of samples. Importantly, here *m* corresponds to the number of genes in a particular signature gene signature and not to all genes that are available in a data set.

### 2.6. Survival Analysis

For evaluating the prognostic value of gene sets, we conduct a survival analysis. Specifically, we estimate a Kaplan–Meier survival curve for each patient group and compare different groups with the Mantel–Haenszel test (Emmert-Streib and Dehmer, 2019). Hence, each comparison is characterized by a *p*-value resulting from a statistical hypothesis test. For the survival analysis, we use the progression-free interval as endpoint.

We would like to remark that due to the fact that the PC1 method provides a categorization of the patients, the resulting survival analysis depends on the gene set used for obtaining the first principal component of the signature.

### 2.7. Measuring of Biological Meaning

In order to have a well-defined meaning of the term “biological meaning,” we use information from the GO (Ashburner et al., 2000). Specifically, GO defines the biological meaning of a gene by a list of GO-terms associated with this gene. For a list of genes, the biological meaning of this set can be defined by the union of the sets of GO-terms of the individual genes. For instance, given three genes, *g*_1_, *g*_2_, *g*_3_, with associate GO-terms the biological meaning (*M*) of these genes is given by

(2) $$\begin{array}{r} M\left(g_{1}\right)=\left\{GO_{1}\left(1\right),GO_{1}\left(2\right),\ldots GO_{1}\left(m\right)\right\} \end{array}$$

(3) $$\begin{array}{r} M\left(g_{2}\right)=\left\{GO_{2}\left(1\right),GO_{2}\left(2\right),\ldots GO_{2}\left(n\right)\right\} \end{array}$$

(4) $$\begin{array}{r} M\left(g_{3}\right)=\left\{GO_{3}\left(1\right),GO_{3}\left(2\right),\ldots GO_{3}\left(o\right)\right\} \end{array}$$

with *m, n, o*∈ℕ. Here, the GO-terms are from a category, e.g., BP. Similarly, the biological meaning of the set of genes {*g*_1_, *g*_2_, *g*_3_} is given by

(5) $$\begin{array}{r} M\left(\left\{g_{1},g_{2},g_{3}\right\}\right)=M\left(g_{1}\right)\cup M\left(g_{2}\right)\cup M\left(g_{3}\right) \end{array}$$

whereas ∪ is the union of the individual sets. Hence, the biological meaning of {*g*_1_, *g*_2_, *g*_3_} is given by the set of GO-terms *M*({*g*_1_, *g*_2_, *g*_3_}).

From this follows that, e.g., the similarity of two sets of genes, {*g*_1_, *g*_2_, *g*_3_} and {*g*_4_, *g*_5_, *g*_6_}, is zero if

(6) $$\begin{array}{r} M\left(\left\{g_{1},g_{2},g_{3}\right\}\right)\cap M\left(\left\{g_{4},g_{5},g_{6}\right\}\right)=\emptyset . \end{array}$$

Importantly, our GRP defined above constructs random gene sets (RGS) with this property, i.e.,

(7) $$\begin{array}{r} M\left(RGS\right)\cap M\left(BM\right)=\emptyset \end{array}$$

with RGS a set of random genes and BM a set of biomarker genes.

## 3. Results

In this section, we present the results of our analysis. First, we study published prognostic biomarkers of PCa individually and comparatively. Then we study random gene set and show results for prognostic outcome.

### 3.1. Prognostic Biomarkers of Prostate Cancer

#### 3.1.1. Size of Biomarker Sets and GO-Terms in Signatures

**Figure 1:**
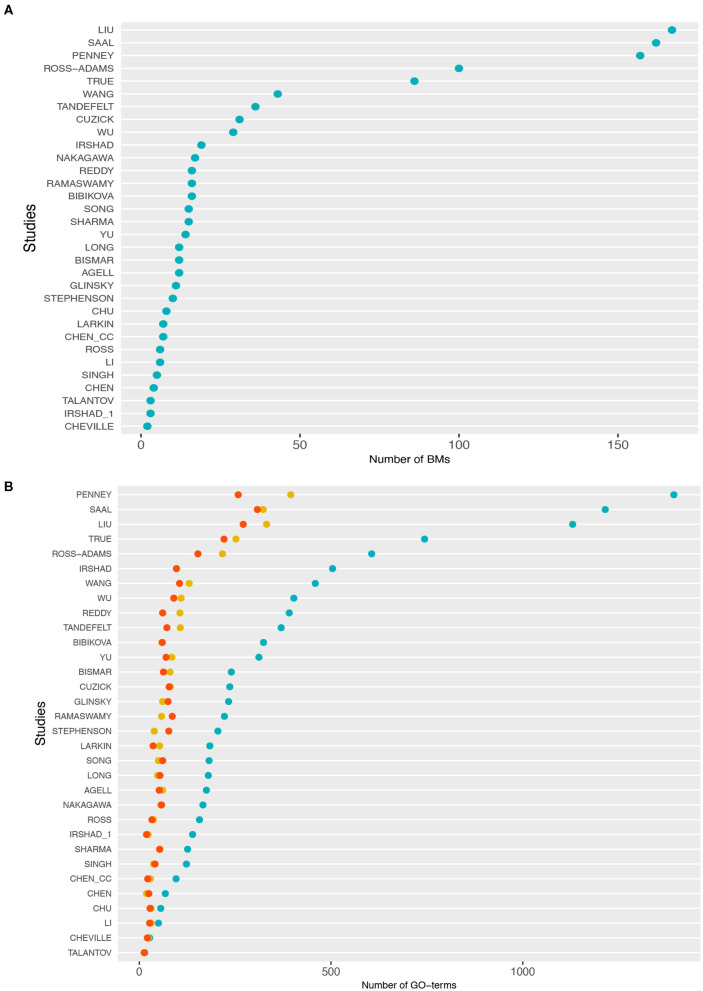
**(A)** Distributions of the number of biomarker genes in each study. **(B)** Number of gene ontology (GO)-terms associated with the signature genes in each study. The cyan points correspond to BP, the red points to molecular function (MF), and yellow points to cellular component (CC).

In Table 1, we show an alphabetically ordered overview of all 32 prognostic BM signatures included in our analysis. The smallest signature is from Cheville consisting of 2 genes only, whereas the signature from Liu is the largest containing 167 genes. Interestingly, there are some signatures that have the same number of genes. Specifically, the signatures of Irshad_1 and Talantov have 3 genes, the signatures of Li and Ross have 6 genes, the signatures of Chen_cc and Larkin have 7 genes, the signatures of Agell, Bismar, and Long have 12 genes, the signatures of Sharma and Song have 15 genes, and the signatures of Bibikova, Ramaswamy, and Reddy have 16 genes in their BM sets. An overall summary of the size distributions of all BM signatures is shown in Figure 1A.

In Figure 1B, we show information about the GO-terms associated with the genes in the signatures. The three colors correspond to the three GO categories: BP shown in cyan, molecular function (MF) shown in red, and cellular component (CC) shown in yellow. For each of these three categories, we show the absolute number of GO-terms in each study. Overall, from Figure 1B one can see that the present GO-terms in the signatures differ significantly from each other. That means some signatures are very specific because they contain only a very small number of different GO-terms, e.g., the signatures from Talantov, Cheville and Li, while others are rather generic containing many GO-terms, e.g., Penney, Liu and Saal. For GO-terms of BP (cyan), this variation is particularly large.

#### 3.1.2. Pairwise Similarity of Signatures

In order to study differences between the 32 signatures, we conduct a pairwise comparison of these BM sets. Specifically, we study two different types of overlap. We study (i) the number of common genes and (ii) the number of common GO-terms among pairs of signatures. Formally, we define these two overlap measures as follows. Let *S*_*i*_ and *S*_*j*_ be two signature sets consisting either of genes or GO-terms. Then we find the percentage *z*_*i*_∈[0, 1] of common elements in *S*_*i*_ that are also present in *S*_*j*_ by

(8) $$\begin{array}{r} x_{i}=S_{i}\cap S_{j} \end{array}$$

(9) $$\begin{array}{r} z_{i}=\frac{\left|x_{i}\right|}{\left|S_{i}\right|} \end{array}$$

Here, *z*_*i*_ can assume values between zero and one and |*z*| corresponds to the number of elements in set *z*. We would like to remark that the way we define the overlap is asymmetric, i.e., *z*_*i*_≠*z*_*j*_ if |*S*_*i*_|≠|*S*_*j*_|. That means the percentage of the overlap is taken with respect to the first signature set *S*_*i*_ on the right-hand side of Equation (8).

**Figure 2:**
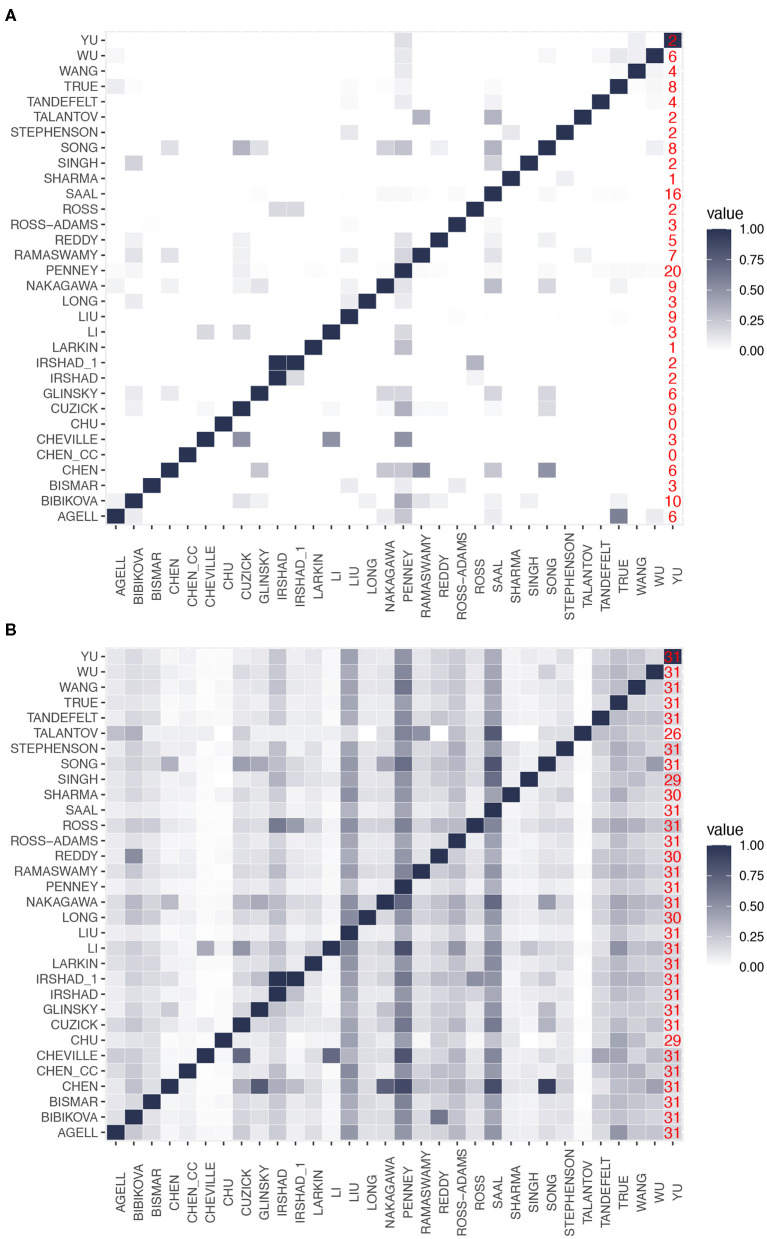
Pairwise overlap between prognostic biomarkers. **(A)** Overlap in the number of genes. **(B)** Overlap in the number of gene ontology (GO)-terms. The last column in both heatmaps (red numbers) gives the number of signatures with a non-zero overlap.

The two heatmaps in Figures 2A,B show the results of this analysis. From this analysis of the gene overlap, we find that the signatures of Chen_cc and Chu do not overlap with other signatures at all, i.e., both have a zero overlap with any other signature. This implies that the genes in the signatures of Chen_cc and Chu are unique concerning the genes in their corresponding BM sets. Every other BM signature has at least some overlap with another signature; see the last column in Figure 2A (red numbers) providing information about the number of signatures with a non-zero overlap.

The signature of Cheville, which has the smallest number of genes, has a gene overlap with the three signatures of Cuzick, Li, and Penney. Surprisingly, the signature of Liu, which contains the highest number of genes, has only genes in common with 9 other signatures. Irshad_1 is the only signature that completely overlaps with another signature (Irshad); however, we would like to note that both signatures are from the same study (Irshad et al., 2013). Finally, we find that the signature of Penney has the highest gene overlap with other signatures (it has genes in common with 20 signatures). From this analysis, we see that there is a wide range of behaviors for the gene overlap reaching from zero overlap (for Chen_cc and Chu) to an overlap with 20 signatures (for Penny) corresponding to an overlap with 64.5% (= 20/31) of all signatures. This implies that all signatures are unique to a certain extend because this percentage would be much higher.

In contrast to these findings, Figure 2B shows the overlap of GO-terms among the signatures. Again, the overlap between the signatures varies considerably. For instance, the signatures of Saal and Penney share the highest overlap with 490 GO-terms. Interestingly, all the signatures have a non-zero overlap in their biological meaning.

Importantly, a difference to the gene overlap (see Figure 2A) is that for a GO-term overlap, all signatures share at least one GO-term with 26 other signatures (see last column in Figure 2B) and most signatures (25) have at least one common GO-term with all other signatures. This implies that on a GO-term level, the signatures are much more similar to each other than on a gene level. This underlines the importance of a systems-view on PCa.

### 3.2. Prediction Abilities of Random Gene Signatures

Next, we systematically investigated the prognostic prediction capabilities of the 32 BM signatures and random gene sets. We begin by systematically removing BM signature genes from the available gene expression gene pool. Subsequently, we also omit hierarchically genes that share a biological meaning with the respective published signatures. We randomly sample 1,000 set of the same size as the BM signature from the gene set left to create random gene signature. The results are as follows:

The outcome of the study is given in three parts. First, from the gene pool, we systematically remove the published signatures and the genes that share a similar biological meaning with them and compute the outcome association. Next, we correct the obtained *p*-values by conservative Bonferroni correction and report the results. And finally, the analysis is repeated by omitting the proliferation genes from the gene pool, we correct the *p*-values by conservative Bonferroni correction, and present the results.

#### 3.2.1. GDC Cohort a Data

**Figure 3:**
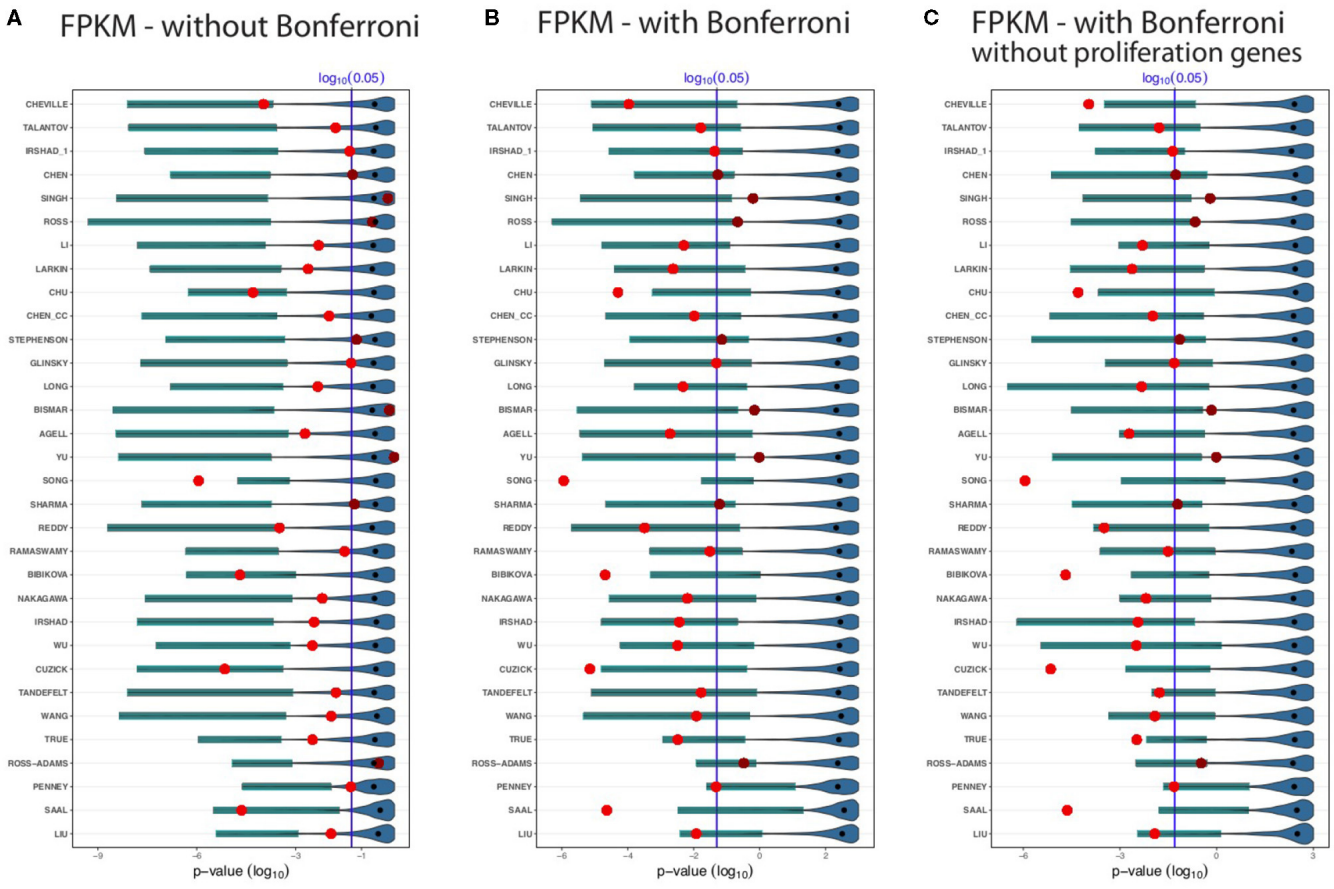
Shown are the prognostic prediction results of random gene sets for 32 signatures using the GDC cohort A data. **(A)** Results for uncorrected *p*-values. **(B)** Bonferroni corrected *p*-values. **(C)** Proliferation genes are removed and the *p*-values are Bonferroni corrected. The significance level is α = 0.05 (vertical blue line) and the light/dark red points represent the outcome of the published signatures. The blue-colored distributions are the results of the random gene sets, whereas the shaded cyan bars correspond to the lower third percentile of the distributions and the bold black points are the median values.

The results for the GDC cohort A data are shown in Figure 3. The light/dark red points represent the outcome of the published signatures (without any gene removal), whereas light red indicates significant results and dark red non-significant one. The blue colored distributions are the result of random gene sets, whereas the shaded cyan bars correspond to the lower third percentile of the distributions and the bold black points are the median values of these distributions. The blue vertical line corresponds to a significant level of α = 0.05. We would like to note that the *p*-values are on a logarithmic scale (i.e., log_10_).

First, from Figure 3 we observed that not all published signatures (red points) lead to significant results. In order to highlight this, we show significant results by points in light red, whereas non-significant results are shown in dark red. A possible reason for this observation is that our analysis uses a different data set than the original studies and, hence, the observed results indicate to the well-known instability of biomarkers (lack of robustness) (Drier and Domany, 2011). Specifically, for our analysis 24 of the 32 biomarker signatures are significant and the remaining published signatures lack robustness for the independent validation data set.

Figure 3A shows results without a Bonferroni correction. This analysis is similar to the study by Venet et al. (2011), which also did not use a multiple testing correction even though many comparisons were conducted. Interestingly, in Figure 3A all lower third percentiles (cyan shaded bars) are significant. That means for all random gene sets we find at least 3% of these to be significant. When compared to the published signatures (red points), the lower third percentile of random gene sets outperform even 26 signatures. Five published signatures performed as well as the lower third percentile of random sets, or the random sets slightly outperformed them. Only one signature (Song) achieves a more significant outcome than the lower third percentile of the random gene sets. Two signatures Ross and Ross-Adams perform as worse as the median of the random sets and three signatures (Singh, Bismar, and Yu) perform even worse than the median of the random gene sets. The median of the random sets (bold black points) are all non-significant.

In Figure 3B, we repeated the analysis applying a conservative Bonferroni correction. With a Bonferroni correction, four signatures (Singh, Ross, Bismar, and Yu) performed worse than the lower third percentile of random signatures. Likewise, five published signatures, Chu, Song, Bibikova, Cuzick, and Saal, outperformed the random signatures. As one can see from Figure 3B, not all the lower third percentile are significant. However, for all random signatures (such as Penney and Liu), we find at least some significant random signatures. Interestingly, many smaller random signatures perform better in comparison to larger ones. For instance, Cheville, Talantov, Irshad_1, Chen, Singh, etc., all performed better than the top 5 largest signatures (True, Ross-Adams, Penney, Saal, and Liu).

In a previous breast cancer study (Goh and Wong, 2018), it has been found that the removal of proliferation genes from random signatures leads to diminishing results of the prognostic performance of random signatures. In order to study this effect, we removed additionally all proliferation genes from the gene pool and repeated our analysis with a Bonferroni correction. The results of this are shown in Figure 3C. Qualitatively, the results in Figures 3B,C are similar. Overall, for all results in Figures 3A–C, one can see that for all random signatures there are at least some that are statistically significant. We would like to emphasize that all random gene sets share *per construction* no biological meaning with the published signatures yet can perform prognosis as well as the BM signatures or better.

#### 3.2.2. GDC Cohort B Data

**Figure 4:**
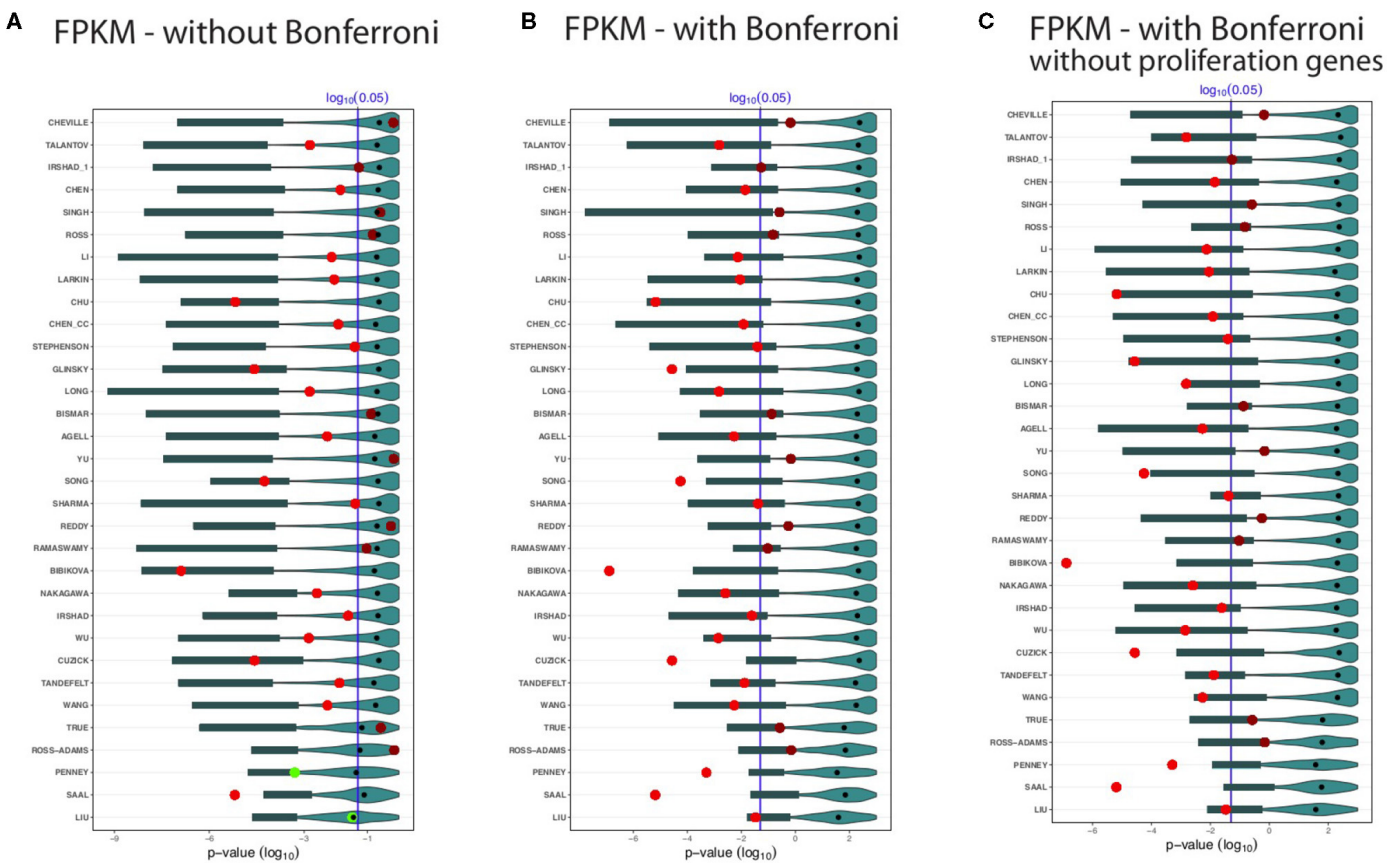
Shown are the prognostic prediction results of random gene sets for 32 signatures using the GDC cohort B data set. **(A)** Results for uncorrected *p*-values. **(B)** Bonferroni corrected *p*-values. **(C)** Proliferation genes are removed and the *p*-values are Bonferroni corrected. The significance level is α = 0.05 (vertical blue line) and the light/dark red points represent the outcome of the published signatures. The blue colored distributions are the results of the random gene sets, whereas the shaded cyan bars correspond to the lower third percentile of the distributions and the bold black points are the median values.

In order to study the influence of the data processing, we repeat our analysis for the GDC cohort B data. The results of this analysis are shown in Figures 4A–C. In these figures, there are in addition to the dark and light red points, light green points indicate the BM signatures. These correspond to significant BM signatures, whereas the median values of the random gene sets (black points) are non-significant.

Again, we observe that not all BM signatures lead to a significant outcome. Specifically, we find 22 of the 32 signatures to be significant (Figure 4). Interestingly, we find also non-robust results. For instance, Cheville, Irshad_1, Reddy, Ramaswamy, and True failed to predict the outcome in the GDC cohort B data set, but these signatures were significant for the GDC cohort A data (see Figure 3). Similarly, Chen, Stephenson, and Sharma are significant for GDC cohort B (see Figure 4) but not GDC cohort A (see Figure 3).

Also for the distributions of the results for the random gene sets, we observe very similar results as for the GDC cohort A data in Figure 3. Hence, overall, the results in Figures 4A–C confirm our analysis, which means there are always random gene sets leading to significant results.

## 4. Discussion

Our hypothesis for the present study was that prognostic signatures of prostate cancer are lacking a sensible biological meaning. In order to investigate this, we used a GRP introduced in Manjang et al. (2021). This GRP allows to systematically construct random gene sets by omitting all biological similarities between published signatures and the genes in a gene pool from which random gene sets are drawn. These random gene sets are not assigned any particular (biological) meaning or role. Importantly, such random gene sets do not necessarily have predictive capabilities as assessed by predicting progression-free survival as outcome variable. For this reason, we distinguish between random gene sets that are predictive (indicated by a significant *p*-value from a survival analysis) and non-predictive by calling the former ones surrogate gene sets. A published BM signature (see Table 1), on the other hand, is a gene set that is obtained in a targeted and non-random manner indicative of disease progression.

For testing our hypothesis, we studied 32 published BM signatures of prostate cancer from the literature (see Table 1). As a result, for all studied 32 signatures we found random gene sets with better or similar prognostic capabilities but no overlap in the biological meaning. In order to see if the preprocessing of the data has any effect on this, we extended our analysis by examining the effect of different data processing techniques. Regarding this, we conducted further analysis by using a data set with different processing methods applied to the raw data leading to a data set we call GDC cohort B. As a result from this analysis, we found no systematic influence of a particular data processing technique on the surrogate gene sets or the overall results (see Figures 3, 4). Finally, we also removed proliferation genes (for both data sets, i.e., GDC cohort A and GDC cohort B) and found also for this setting no difference in our results (see Figures 3C, 4C).

As a conclusion from all these analyses, we can infer that any biological rationale provided for selecting the genes in the published gene signatures, as shown in Table 1, is anecdotal. This is taking into account the meaning of random gene sets arising from the GRP because the used GRP eliminates the risk of accidentally selecting genes for a random gene set that have the same biological meaning as the published gene signatures. Consequently, due to the discovery of surrogate gene sets with the same predictive capability but a completely distinct biological interpretation, as a result of the zero overlap in the GO-terms of the genes involved, any biological significance attributed to such BM signatures is required.

Interestingly, a similar interpretation has been found in a breast cancer study by Manjang et al. (2021). They showed that when all signs of the biological meaning of the BM signature genes are removed, surrogate gene sets can be determined among the remaining random gene sets with similar prognostic predictive capabilities but with contrasting biological meaning. Therefore, the research findings indicated that with regard to disease etiology, none of the studied signatures have a plausible biological interpretation or significance. The study concluded that prognostic signatures are black-box models that can yield accurate predictions of breast cancer outcome but with no benefit for disclosing causal, biological relations. Furthermore, this study also noted a relationship between the predictive accuracy and the size of the random gene sets by showing that the accuracy is higher for larger gene sets. It is interesting to note that in the current study, we could not establish this relationship. A possible explanation for this may be the relatively small size of published BM signatures of prostate cancer, which are all smaller than 200 genes (see Figure 1). In contrast, the breast cancer signatures studied in Manjang et al. (2021) are much larger in average reaching up to 1345 genes.

It is important to note that a similar study for breast cancer by Venet et al. (2011) has been unable to arrive at this conclusion since no GRP was used. As a consequence, BM signatures as well as genes from associated BP were not removed leaving the possibility to inadvertently select random genes with a common biological meaning as the original BM signatures because these genes belong to the same BPs as indicated by common GO-terms in the domain BP. Another statement by Venet et al. (2011) is that *most random signatures are significantly associated with prognostic outcome*. With respect to prostate cancer, this holds only for the random gene sets of Penney and Liu (see Figure 4A) because 50% of the surrogate gene sets are significant as indicated by the median values of the distributions (black points in Figure 4). However, generally, this assertion is not valid and only applies to some signatures.

To date, many studies investigated prognostic signatures of prostate cancer. For example, Bibikova et al. (2007) used a 16-gene expression signature to predict the prognosis of prostate cancer. They complemented their results by a discussion of the functional annotation of these genes, which were involved in proliferation, cell cycle, differentiation, signal transduction and basic metabolism. Similarly, the studies by Saal et al. (2007), Sharma et al. (2013), and Song et al. (2019) argued that the biological importance of their prognostic signatures is based on the role of PI3K pathway signaling, altered signaling, P53 signaling and cell cycle process pathway respectively. In this paper, we studied those and other prognostic signatures of prostate cancer. Our results, however, demonstrate that such biological interpretations do not offer a causal explanation for the fundamental biology of prostate cancer since we can always find surrogate gene sets with no biological relationship to those signatures but similar or better prognostic prediction capabilities.

Considering that prostate cancer and breast cancer are two considerably different diseases yet our results demonstrate a similarity in the lack of biological meaning of both cancers one may wonder if there is a common factor giving raise to these findings. This is very difficult to answer, however, one common factor that comes to mind are the hallmarks of cancer (Hanahan and Weinberg, 2000). Specifically, the study by Hanahan and Weinberg (2000) highlighted six hallmarks of cancer (self-sufficiency in growth signals, insensitivity to growth-inhibitory (antigrowth) signals, evasion of programmed cell death (apoptosis), limitless replicative potential, sustained angiogenesis, and tissue invasion and metastasis), which are shared by all types of human cancers. Later this has been extended by four further hallmarks (deregulating cellular energetics, avoiding immune destruction, genome instability and mutation, tumor-promoting inflammation) (Hanahan and Weinberg, 2011). If our findings are actually related to the ten hallmarks of cancer is currently unclear. However, it seems not implausible to assume that there might be a connection because the hallmarks state that cancer is a system disease involving a multitude of pathways. We want to add that these pathways do not work in isolation but are connected among each other by intricate regulatory networks (Emmert-Streib et al., 2014).

On a technical note, we would like to remark that there could be other metrics for evaluating the prediction capabilities of random gene sets other than *p*-values. For instance, one could use information from pathology about disease states, which allow to use error measures for binary classifications. While this establishes sensible metrics, e.g., F-score or AUROC, such measures do not directly utilize survival information about the progression of patients. Instead, this is the strength of survival analysis comparing survival curves quantitatively. Hence, a regression framework, as provided by survival analysis (Kleinbaum and Klein, 2005), seems to be favorable over a classification framework allowing a more nuanced evaluation.

Finally, we would like to note that our study has similarities to recent investigations in Explainable Artificial Intelligence (XAI) (Xu et al., 2019; Emmert-Streib et al., 2020). Specifically, XAI explores the dichotomy of predictive and descriptive models (Emmert-Streib and Dehmer, 2021) in AI and aims to establish mechanisms for making predictive models also explainable in a sense that this can enhance our understanding of a system under investigation. On a wider scope, this discussion has a long history in the statistics community and refers to the distinction of black-box models and causal models (Holland, 1986; Breiman, 2001). Our study shows that prognostic biomarkers of prostate cancer allow sensible predictions for cancer progression but do not establish a causal understanding with respect to the biological meaning of such prognostic signatures. Here, it is important to extend the considerations to the proposed gene selection mechanisms used by studies identifying prognostic signatures (see Table 1). Overall, such models have a predictive utility, e.g., for applications in the clinical practice but no biological utility for enhancing our understanding of cancer biology.

## 5. Conclusion

In this paper, we scrutinized the biological meaning of prognostic signatures of prostate cancer. Our study utilized a GRP that results in random gene sets without any overlap in the biological meaning with biomarker signatures yet a non-vanishing proportion of these random gene sets, called surrogate gene sets, achieve similar prediction results. Hence, our results demonstrate that none of the studied signatures of prostate cancer has a sensible biological interpretation with respect to disease etiology. To our knowledge, this is the first study providing such results for prognostic biomarkers of prostate cancer.

## Data Availability Statement

Publicly available datasets were analyzed in this study. This data can be found here: https://xenabrowser.net/datapages/.

## Author Contributions

FE-S conceived the study. KM performed the analysis. KM and FE-S analyzed the data and interpreted the results. All authors wrote the manuscript.

## Conflict of Interest

The authors declare that the research was conducted in the absence of any commercial or financial relationships that could be construed as a potential conflict of interest.
